# Tim-3 Is Upregulated in NK Cells during Early Pregnancy and Inhibits NK Cytotoxicity toward Trophoblast in Galectin-9 Dependent Pathway

**DOI:** 10.1371/journal.pone.0147186

**Published:** 2016-01-20

**Authors:** Jintang Sun, Meixiang Yang, Yanli Ban, Wenjuan Gao, Bingfeng Song, Yang Wang, Yun Zhang, Qianqian Shao, Beihua Kong, Xun Qu

**Affiliations:** 1 Institute of Basic Medical Sciences, Qilu Hospital, Shandong University, Jinan, Shandong, People's Republic of China; 2 Department of Obstetrics and Gynecology, Qilu Hospital, Shandong University, Jinan, Shandong, People's Republic of China; Xavier Bichat Medical School, INSERM-CNRS - Université Paris Diderot, FRANCE

## Abstract

NK cells accumulate at the maternal-fetal interface (MFI) and play essential roles in maintaining immune tolerance during pregnancy. The mechanisms that facilitate NK cells tolerance to fetal tissue are largely unknown. T cell Ig and mucin domain-containing protein 3 (Tim-3) is a newly defined molecule with essential immunological function in many physiological and pathological processes. Recent study showed that Tim-3 was involved in the regulation of immune tolerance at MFI. However, whether Tim-3 regulates NK cells cytotoxicity toward trophoblasts is unclear. Here, we showed Tim-3 was mainly expressed by decidual NK cells (dNK) and Tim-3 level in dNK was higher than peripheral NK cells (pNK). Tim-3^+^ dNK expressed more levels of mature markers CD94 and CD69 than Tim-3^-^ dNK cells and blocking Tim-3 significantly inhibited dNK IFN-γ and TNF-α secretion. Furthermore, we found TGF-β1 may contribute to such up-regulation of Tim-3 in NK cells. Interestingly, blocking Tim-3 enhanced NK cytotoxicity toward trophoblast cell line HTR-8 but not K562. We found HTR-8 expressed Tim-3 ligand Galectin-9, in contrast K562 did not. Small interfering RNA-mediated silencing of Galectin-9 expression enhanced NK cytotoxicity toward HTR-8. We further showed Tim-3/Galecin-9 inhibited NK cytotoxicity toward trophoblast partially via impairing the degranulation process. In addition, clinical data showed that abnormal Tim-3 level on pNK might be associated with recurrent spontaneous abortion (RSA). Thus, our data demonstrate Tim-3/Galectin-9 pathway maintains local tolerance by suppressing NK cytotoxicity toward trophoblasts which may represent a new immunologic tolerance mechanism at MFI.

## Introduction

Maternal immune tolerance to semiallograft fetus is prerequisite for normal pregnancy outcome and represents a great unsolved issue of immunology [1, 2]. One prominent feature of the pregnant human decidua is the dramatical accumulation of NK cells, which constitute 70% of the total leukocytes in the first trimester of pregnancy [3]. In contrast to peripheral NK (pNK) cells, human decidual NK (dNK) cells express high levels of CD56 and lack the expression of CD16 and represent a unique NK cell subset with immunomodulatory role in implantation and pregnancy [4, 5]. It has been shown that although dNK cells are granular and express the essential molecules required for lysis, freshly isolated dNK displayed about 15% lytic activity of that of pNK [6]. However, recent studies showed that dNK cells might switch on their cytotoxicity, leading to fetal resorption or preterm birth in IL-10^-/-^ mice exposed to LPS [7, 8]. Other studies also demonstrated that the cytotoxicity of dNK cells toward target cells, including trophoblasts, could be promoted when cultured in vitro with IL-2 [9, 10]. Furthermore, increased cytolytic NK cells have been detected in the endometrium of patients with a history of RSA and implantation failure [11]. These results suggested that cytotoxicity of dNK toward trophoblasts should be tightly regulated during pregnancy. But the mechanisms that facilitate pregnancy-compatible, noncytotoxic characteristics of dNK cells need to be further delineated.

Tim-3 was initially identified as a negative regulator of Th1 immunity and shown to induce T cell exhaustion in chronic viral infection and cancers after ligation of Galectin-9 [12, 13]. In contrast, NK cells expressed the highest amounts of Tim-3 among lymphocytes, and the level of Tim-3 in NK cells can be further up-regulated on activation [14]. Lishomwa C. Ndhlovu et.al showed that Tim-3 marked highly functional NK cells with respect to both cytokines production and degranulation [14]. Consistently, Michelle K. Gleason et.al demonstrated that Tim-3 was a coreceptor of NK cells to enhance IFN-γ production [15]. However, when Tim-3 was cross-linked with antibodies it suppressed NK cell mediated cytotoxicity [14]. Furthermore, recent data indicated that Tim-3 functioned as a exhaustion marker of NK cells in advanced melanoma [16] and negatively regulated NK function in LPS-induced endotoxic shock [17]. So, the roles of Tim-3 in regulating NK cells function are controversial.

Notably, it has been reported that systemic blockade of Tim-3 leads to abrogation of MFI tolerance and fetal rejection in mouse model [18]. In human, Tim-3 is strikingly upregulated in peripheral monocytes and abnormal Tim-3 expression on peripheral monocytes might be connected to RSA [19]. Furthermore, Evo Miko et. al showed that Tim-3 levels on T cells and NK cells were significantly decreased in early-onset preeclampsia patient compared to healthy pregnant women [20]. Li YH et.al demonstrated that dNK cells expressed Tim-3 and a decreased percentage of Tim-3 positive dNK cells were detected in human miscarriages and murine abortion-prone model [21]. All these reports indicate Tim-3 may have essential roles in pregnancy. However, whether Tim-3 regulates NK cells cytotoxicity against trophoblasts has remained largely unknown. In this paper, we found dNK cells was the major immune cell that expressed Tim-3 at MFI and dNK expressed more level of Tim-3 than pNK cells. Tim-3 inhibited NK cells cytotoxicity toward trophoblasts in Galectin-9 dependent pathway partially via impairing the degranulation process. In addition, we showed that pNK cells from RSA patient expressed lower level of Tim-3 than normal pregnancy control. Our findings suggest a mechanism by which NK cytotoxicity toward trophoblasts is inhibited, which promotes immune tolerance at MFI.

## Materials and Methods

### Ethics Statement

The collection and use of blood and decidual sample complies with relevant guidelines and institutional practices from the Ethics Committees of Qilu Hospital of Shandong University and the written approval was obtained in each case before blood and decidual sample collection. Our study was specially approved by Ethics Committees of Qilu Hospital of Shandong University (No. KYLL-2013-069).

### Reagents

RPMI-1640 medium was purchased from Hyclone (Utah, USA) and Fetal Bovine Serum was from Bio International (Auckland, New Zealand). rhTGF-β1 and rhGalectin-9 were from R&D (Minneapolis, USA). Goat anti-human TGF-β1, goat anti-human Galectin-9 and Tim-3-Fc fusion protein were purchased from R&D Systems. Fluorescently-labeled antibody for FACS analysis, CD3, CD56, CD16, Tim-3, CD117, CD94, CD11b and CD69 were purchased from BD PharMingen (San Diego, California). CD107a was from Miltenyi Biotec.

### Subjects

Peripheral blood and decidual samples were collected from first-trimester pregnant women (from 8–12 weeks) who had undergone elective termination of normal pregnancy. Blood samples were also collected from unexplained RSA patients and non-pregnant healthy women. All subjects ranged from 22 to 35 years old and none of them had shown any pathological manifestations, such as uterine abnormalities, infections or pre-existing diseases.

### Cell lines

Placental trophoblasts cell line HTR-8/SVneo (HTR8) was kindly donated by Dr Charles H. Graham (Queen’s University, Ontario, Canada) [22]. HTR-8 and K562 (obtained from ATCC) were cultured in RPMI 1640 medium, supplemented with 10% FCS, 100 U/ml penicillin and 100 μg/ml streptomycin.

### Isolation of NK cells from peripheral blood and decidua

Peripheral blood mononuclear cells (PBMC) were obtained by centrifugation with Ficoll-Paque Plus (Amersham Biosciences, Piscataway, NJ). CD56^+^ cells from PBMC were enriched with a bead-labeled anti-CD56 monoclonal antibody using the magnetic antibody cell sorting (MACS) system (Miltenyi Biotec). CD56^+^CD3^-^ NK cells from decidua were isolated using BD Influx Cell Sorting System following the manufacturer’s instructions (Becton Dickinson, CA). The purity of CD56^+^ cells was consistently over 95%.

### Flow cytometric analysis

PBMC or decidual lymphocytes were stained with appropriate fluorescence conjugated mAb or isotype controls for 20 min away from light. Then cells were washed with cold PBS for total three times, the expression of surface molecules were assayed by FACS (Calibur, Becton Dickinson, CA). A minimum of 10 000 events per sample were collected for phenotypic analysis. For degranulation assay, freshly isolated NK cells were pretreated with rhTGF-β1 and then were cultured in complete media or stimulated with HTR-8 cells (10:1 effector: target ratio) in the presence of APC-conjugated anti-CD107a mAb and monensin (eBioscience) for 3 hours. The cells were then harvested and washed for CD107a assay by FACS.

### Real-time quantitative RT-PCR

Total RNA was prepared using the RNeasy mini kit and purified using RNeasy mini spin columns (Qiagen Inc., Valencia, CA, USA) according to the manufacturer’s protocol. RNA concentration was quantified by UV spectrophotometer at 260 nm and the purity and integrity was determined using the A260/A280 ratio and lab-on-chip assay (Agilent bioanalyzer). For reverse transcription, cDNA was synthesized with oligo dT primers and Moloney Murine Leukemia Virus Reverse Transcriptase (MMLV-RT) according to the instructions (Invitrogen). Real-time PCR was performed on a LightCycler 2.0 Instrument (Roche, Penzberg, Germany) using Fast Start DNA Master SYBR Green I kit (Roche). All the procedures were conducted according to the manufacturer’s protocols. The primer sequences were as follow, *Tim-3*: sense, 5'-ACTTCACTGCAGCCTTTCC-3', anti-sense, 5'-CGAATTCCCTCTGCTACTGC-3'; *Galectin-9*: sense, 5'-TGCAACACGAGGCAGAACG-3', anti-sense, 5'-CACAGAGCCATTGACGGAGAT-3'; *TGF-βRI*: sense, 5'-TGGCGATACCTCAGCAACC-3', anti-sense, 5'-CTCGTGGATCCACTTCCAG-3'. *β-actin*: sense, 5'-TTGCCGACAGGATGCAGAA-3', anti-sense, 5'-GCCGATCCACACGGAGTACT-3'. Levels of the housekeeping gene β-actin were used as an internal control for the normalization of RNA quantity and quality differences among the samples. Gene-specific amplifications were demonstrated with melting curve data.

### Immunohistochemical staining

Formalin fixed paraffin-embedded decidual tissue sections were stained with Goat anti-human TGF-β1 and Galectin-9 and biotinylated rabbit anti-goat Ig (Maixin Co., Fuzhou, China) followed by streptavidin-conjugated peroxidase. DAB was used as substrate.

### Cytokine measurement

Contents of cytokines were evaluated using commercially available multiplex bead-based sandwich immunoassay kits by Bio-Plex Protein Array system (Bio-Rad Laboratories, USA) following the manufacturer’s instructions. For each cytokine, eight standards with the concentrations in the range of 0.2 to 3200 pg/mL were used and the minimum detectable dose was <1 pg/mL. A volume of 50 μl was sampled from each well and the fluorescent signal of a minimum of 100 beads per region was evaluated and recorded.

### Cytotoxicity assays

Freshly isolated pNK cells from non-pregnant healthy women were pretreated with rhTGF-β1 (2ng/ml) and used as effector cells. HTR-8 or K562 cells were used as target cells. Target cells were pre-incubated with human Tim-3-Fc fusion proteins (RD) for 30 min before co-cultured with the effector cells at indicated ratios. Four hours later, cells were stained with a Cell Counting Kit-8 (CCK-8) (Dojindo, Kumamoto, Japan) and the absorbance at 450 nm of each well was measured according to the manufacturer’s instructions. All experiments were done in triplicates.

### Statistical analyses

Data are presented as mean±SD. One-way Anova was used for statistical analysis, and a *p* value of < 0.05 was considered statistically significant.

## Results

### High expression of Tim-3 by decidual NK cells

**Fig 1:**
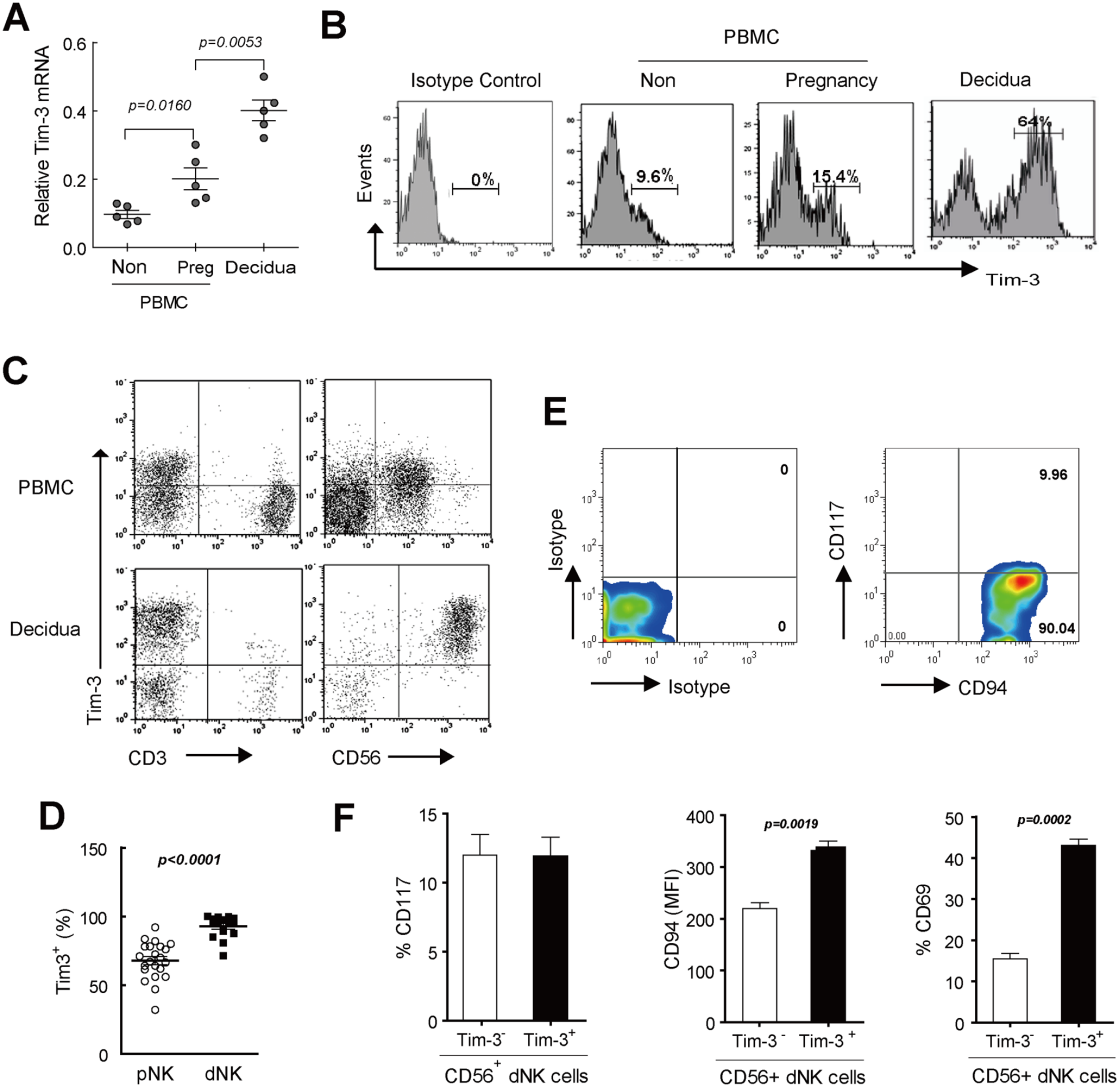
dNK expressed high level of Tim-3 during early pregnancy. The expression of Tim-3 in PBMC from early pregnant women (n = 5) and non-pregnant control (n = 5) and decidual lymphocytes of early pregnant women (n = 5) was detected by real-time PCR (A) and FACS (B), one representative result was shown. (C), The Tim-3 was mainly expressed by CD3^-^CD56^+^ pNK and dNK cells according to flow cytometric analysis, one representative result was shown. (D) The expression of Tim-3 on dNK cells (n = 15) and pNK cells (n = 22) was determined by flow cytometry. (E) Lymphocytes were isolated from decidua as described in materials and methods and stained with fluorescence conjugated CD56, CD117, CD94 and isotype control mAb. CD117 and CD94 expression on CD56^+^ NK cells was determined by FACS, one representative result was shown, n = 5. (F) Statistical analysis of the expression of CD117, CD94 and CD69 on Tim-3^-^ and Tim-3^+^ dNK cells (n = 5). MFI, Mean Fluorescence Intensity.

To delineate the contribution of Tim-3 to pregnancy immunology, we examined its mRNA and surface levels in PBMC and decidual lymphocytes by RT-PCR and FACS respectively. As shown in Fig 1A and 1B, PBMC from pregnant women showed higher Tim-3 mRNA and surface protein expression than non-pregnant women control and decidual lymphocytes expressed much more Tim-3 than corresponding PBMC. Furthermore, we found Tim-3 was mainly expressed by CD3^-^CD56^+^ NK cells both for PBMC and decidual tissue (Fig 1C). In contrast, only a few CD3^+^ T cells were positive for Tim-3. B cells did not express Tim-3 (data not shown) which was consistent with other report^19^. So we further compared Tim-3 level on dNK and corresponding pNK cells. Our results showed that nearly 90% dNK cells were Tim-3 positive which was significantly higher than the level in pNK cells (Fig 1D).

Although Tim-3 is considered as a maturation marker of pNK, the connection between Tim-3 expression and dNK differentiation and maturation is totally unclear. We found that freshly isolated dNK cells were mainly CD117^-^CD94^+^ mature stage NK cells (Fig 1E) which is consistent with other report^23^. Tim-3^+^ dNK cells expressed high level CD94 than Tim-3^-^ dNK cells, but CD117 level on these two NK groups had no difference (Fig 1F). Meanwhile, the activation receptors CD69 in Tim-3^+^ dNK cells were also higher than Tim-3^-^ dNK cells (Fig 1F). These results suggested that Tim-3^+^ dNK represented a more mature NK subtype with much more activation.

### Tim-3 signal modulated the cytokine production of decidual NK cells

**Fig 2:**
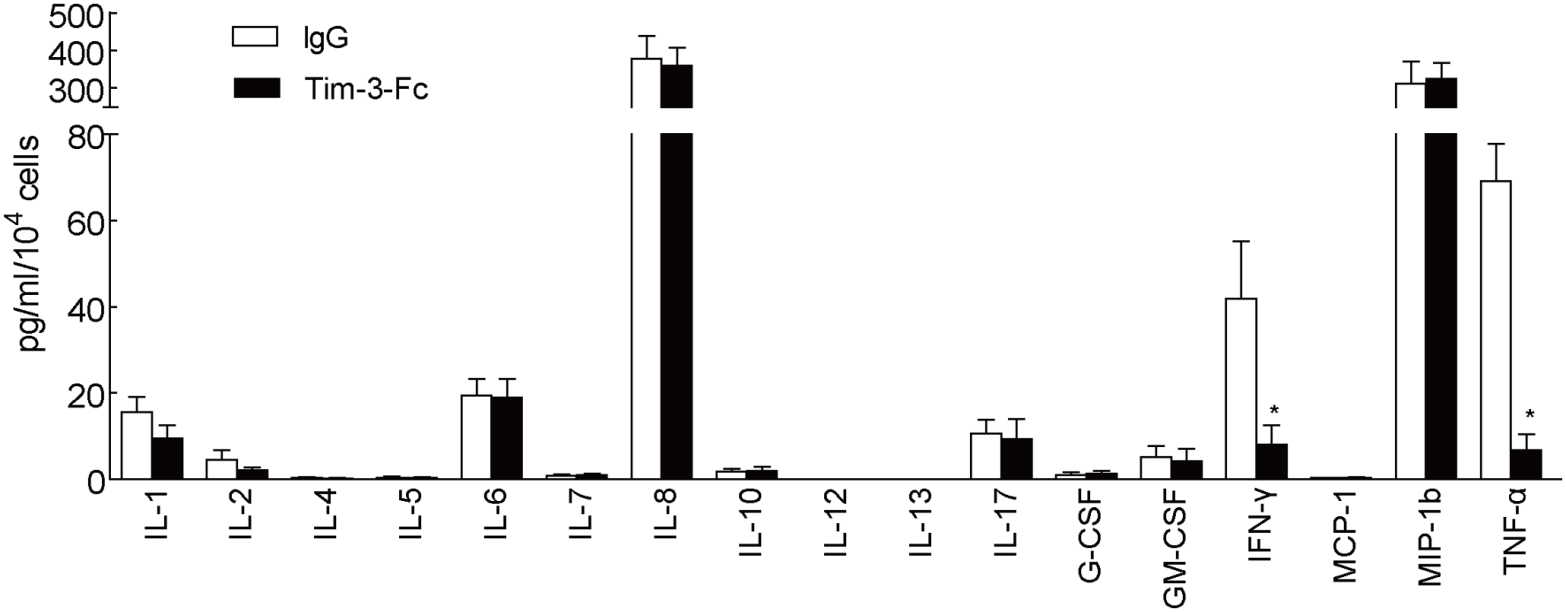
Tim-3 modulated the cytokine production of decidual NK cells. Isolated CD56^+^ dNK cells were cultured for 24 hours in the presence or absence of Tim-3-Fc fusion proteins with low does rhIL-2 (50U/ml). Supernatants were harvested and the cytokine production was quantified using multiplex bead-based sandwich immunoassay kits. Data are expressed as the mean ± SD of four independent experiments. *, *p*< 0.05.

By using multiplex bead-based sandwich immunoassay kits, we measured the cytokine production of dNK cells. The results showed that dNK cells could express a variety of cytokines, including IL-1β, IL-6, IL-8, IL-17, GM-CSF, IFN-γ, MIP-1β, and TNF-α (Fig 2). Blocking Tim-3 signal with Tim-3-Fc fusion protein could significantly inhibit IFN-γ and TNF-α production (Fig 2). But other cytokines including IL-6, IL-8 and MIP-1β were not regulated by Tim-3. Thus, these results suggest that the increased expression of Tim-3 could contribute to the cytokine production of dNK cells.

### Tim-3 inhibited NK cytotoxicity toward trophoblasts

**Fig 3:**
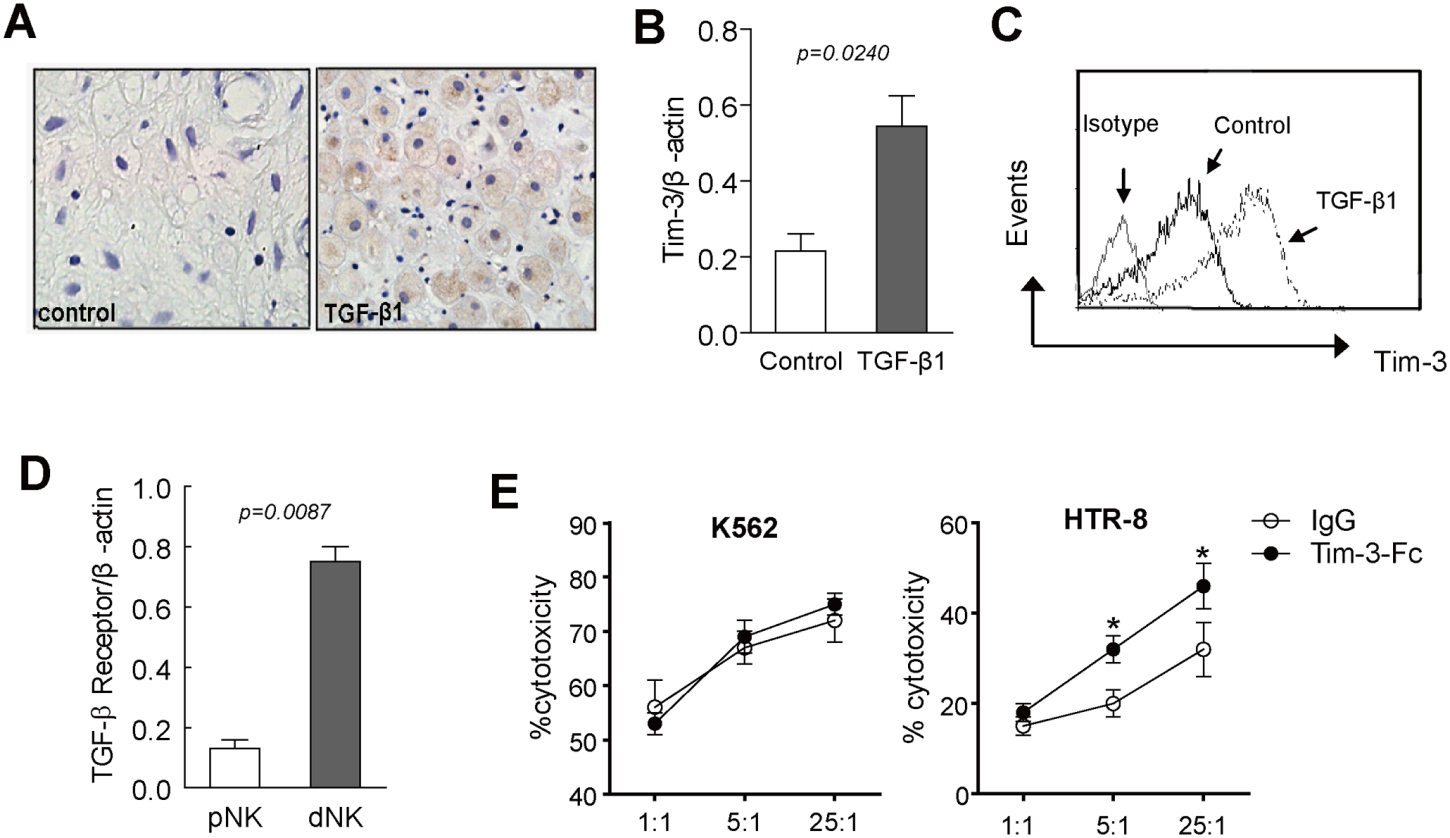
Tim-3 inhibited cytotoxicity of NK cells toward trophoblasts. The expression of TGF-β1 in early pregnant decidua was detected by immunohistochemical staining (A). CD56^+^ NK cells were isolated from PBMC of healthy non-pregnant women (n = 5) as described in the material. The expression of Tim-3 was detected after NK cells were stimulated with TGF-β1 (2ng/ml) by RT-PCR (B) and FACS (C), one representative result was shown. *, *p* < 0.05. Control, isotype control antibody group. (D) The expression of TGF-βRI was detected by real-time PCR in early pregnant women pNK and dNK cells. *, *p* < 0.05. *E*, Target cells (K562 or HTR8) were pre-incubated with Tim-3-Fc fusion proteins or isotype control IgG for 30 min and then co-cultured with TGF-β1 pretreated pNK cells from healthy control (n = 5) at different E:T ratio for 4 hours. The cytotoxicity of NK cells was measured using Cell Counting Kit-8.

TGF-β1 plays vital role in pregnancy and it has been reported that TGF-β1 induced conversion of pNK to a dNK like phenotype [23]. Firstly, we confirmed the expression of TGF-β1 in decidual tissue by immunohistochemical staining (Fig 3A). Then, we want to know whether TGF-β1 mediated conversion of pNK to dNK followed by Tim-3 upregulation. We stimulated isolated pNK cells in vitro with rhTGF-β1 and the result showed that both Tim-3 mRNA and surface levels were significantly up-regulated (Fig 3B and 3C). Notably, dNK cells expressed higher level of TGF-βRI than that in pNK cells (Fig 3D), which may be associated with the difference of Tim-3 level between dNK and pNK cells. Taken together, these data suggested that pregnancy-related immune regulator TGF-β1 may be involved in Tim-3 expression in NK cells during pregnancy. We next evaluated the role of Tim-3 on NK cytotoxicity. For this, we used TGF-β1 induced dNK like cells as effector cells and we found TGF-β1 treatment significantly inhibited NK cytotoxicity toward targets including a NK sensitive cell line K562 and trophoblasts cell line HTR-8 (data not shown). Furthermore, Tim-3-Fc had no effect on NK cytotoxicity toward K562. However, NK cytotoxicity toward HTR8 was significantly enhanced in the presence of Tim-3-Fc (Fig 3E).

### Tim-3 inhibited NK cytotoxicity toward trophoblasts in Galectin-9 dependent pathway

**Fig 4:**
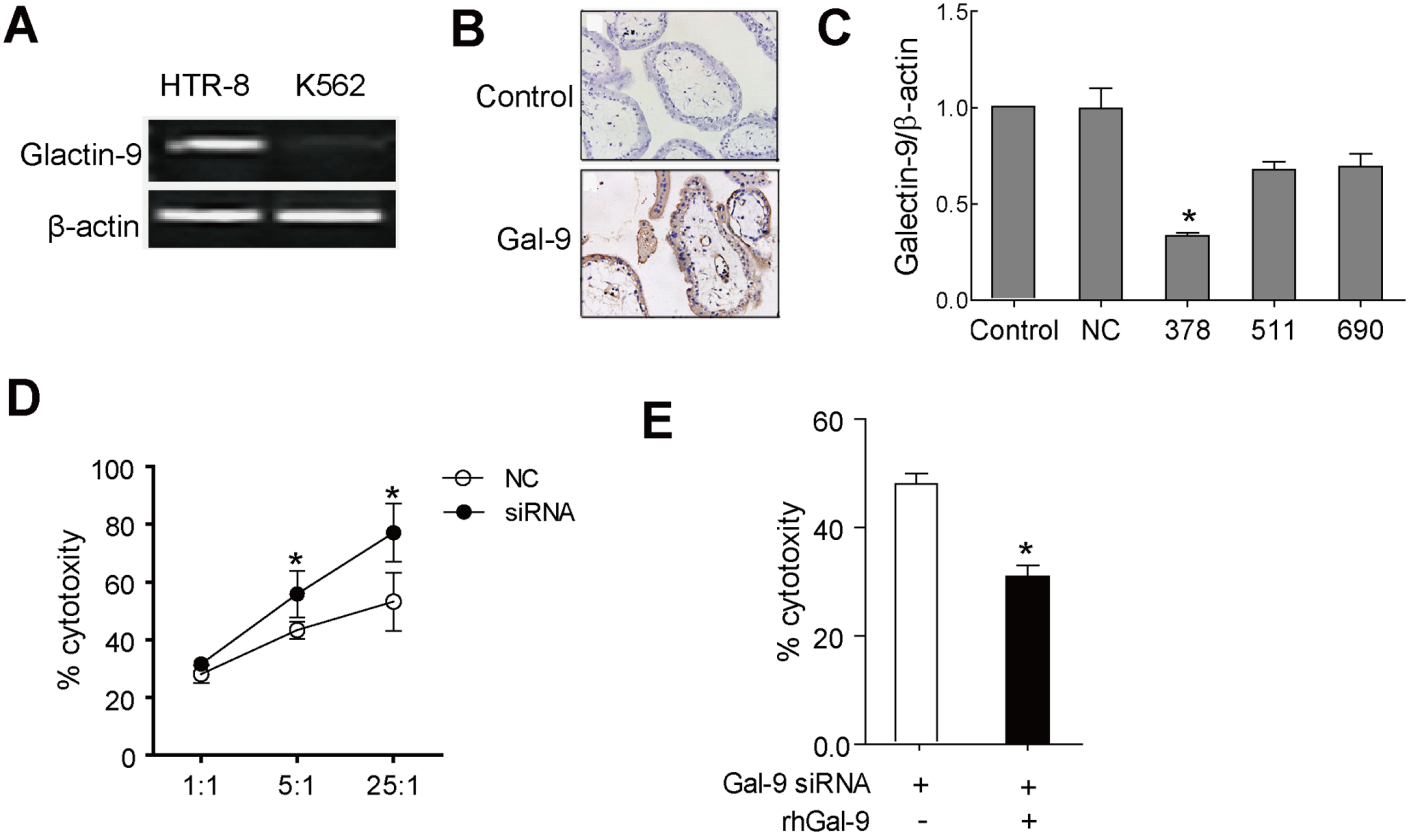
Tim-3 inhibited cytotoxicity of NK cells toward trophoblasts in Galectin-9 dependent manner. (A) The expression of Galctin-9 in HTR8 and K562 cells was determined by RT-PCR. (B) The expression of Galectin-9 in human early pregnant trophoblasts cells was detected by immunohistochemical staining (×400). Formalin fixed paraffin-embedded trophoblasts tissue sections were stained with Goat anti-human Galectin-9 and biotinylated rabbit anti-goat Ig followed by treptavidin-conjugated peroxidase. (C) Knock down Galectin-9 expression by siRNA. The knock down efficacy was determined by RT-PCR. (D) Galectin-9 siRNA significantly increased NK cytotoxicity toward trophoblasts. HTR-8 cells, transfected with NC control or Galectin-9 siRNA, were used as targets and co-cultured with TGF-β1 pretreated pNK cells. The cytotoxicity of NK cells was measured using a Cell Counting Kit-8. (E) HTR-8, transfected with Galectin-9 siRNA, and co-cultured with TGF-β1 pretreated pNK cells at E:T ratio (10:1) with or without rhGalectin-9 (rhGal-9) for 4 hours. The cytotoxicity of NK cells was measured using a Cell Counting Kit-8.

It has been reported that the regulation of Tim-3 on NK cytotoxicity depended on its ligand level in the local microenvironment. To further substantiate the differences between NK cytotoxicity toward different targets, we detected the expression of Tim-3 ligand Galectin-9 in HTR8 and K562 cells respectively and found that Galectin-9 was highly expressed in HTR-8 cells, but not in K562 cells (Fig 4A). Consistently, immunohistochemical staining showed high Galectin-9 expression in decidual tissue (Fig 4B). These results suggested that Tim-3 may inhibit the cytotoxicity of NK cells in Galectin-9 dependent pathway. To test this, we used small interfering RNA to knock down Galectin-9 in HTR-8. Based on the results, siRNA-378 had the best inhibitory effect on Galectin-9 expression (Fig 4C), so we selected siRNA-378 to do the following experiments. As shown in Fig 4D, treatment with Galectin-9 siRNA resulted in enhancement of NK cytotoxicity against HTR-8. In addition, the regulation of cytotoxicity through Tim3 was restored in the presence of rh-Glactin-9 (Fig 4E).

### Tim-3 impaired NK degranulation process via Galectin-9

**Fig 5:**
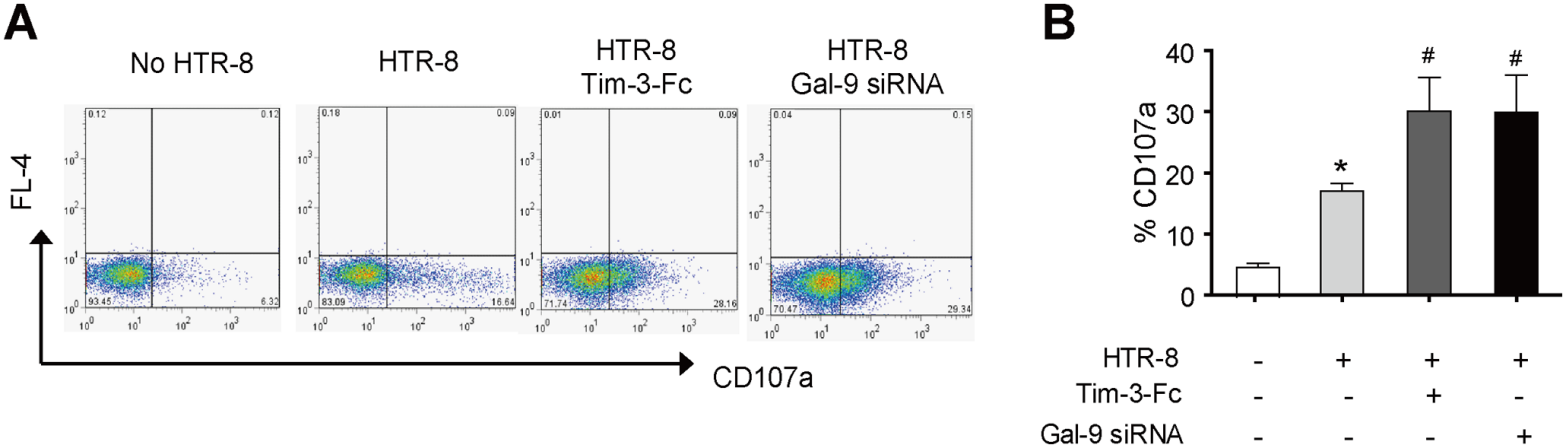
Tim-3-Galectin-9 pathway regulated NK degranulation process. NK cell alone or co-cultured with HTR-8, HTR-8 treated with Tim-3-Fc or HTR-8 transfected with Galectin-9 siRNA for 3h. Then the co-cultures were harvested and incubated with CD56-FITC. The level of CD107a on CD56^+^ cells was determined by FACS, one representative result was shown (A). (B) Statistical analysis of CD107a expression (n = 5). *, *p*< 0.05 compared with no HTR-8 stimulation group, #, *p*< 0.05 compared with HTR-8 stimulation group.

Degranulation is an essential process for NK cells cytotoxicity. As expected, we showed that co-cultured with HTR-8 significantly increased NK cells degranulation process determined by CD107a expression (Fig 5A). Tim-3 blockage with Tim-3-Fc antibody predominantly increased CD107a expression (Fig 5A and 5B). Furthermore, CD107a level was also significantly upregulated when HTR-8 cells were treated with siRNA of Galectin-9 (Fig 5A and 5B). Thereafter, these results suggested that Tim-3-Galectin-9 pathway inhibited NK cells cytotoxicity toward trophoblasts partly via regulating degranulation process of NK cells.

### A possible relevance between Tim-3 abnormality and unexplained RSA

**Fig 6:**
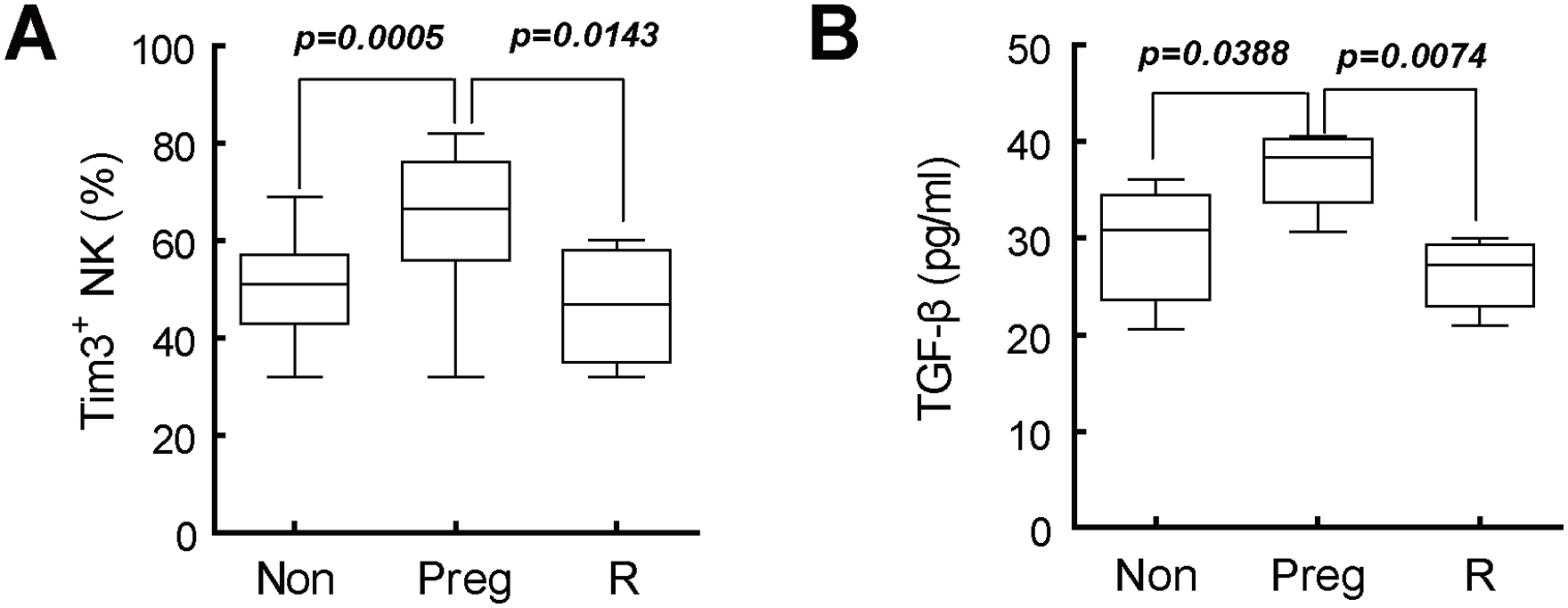
The expression of Tim-3 was abnormally in unexplained RSA patients. (A) The expression of Tim-3 in CD3^-^CD56^+^ pNK cells of non-pregnant women (Non, n = 30), early pregnant women (Preg, n = 30) and unexplained RSA patients (R, n = 20) was analyzed by flow cytometry. (B) Serum level of TGF-β1 in non-pregnant women, early pregnant women and unexplained RSA patients was detected by ELISA.

To discover the possible clinical significance of Tim-3, we analyzed the expression of Tim-3 on pNK cells from normal pregnant and RSA patients. As shown in Fig 6A, the level of Tim-3 in RSA patients was lower than that of the normal pregnant women. As the expression level of Tim-3 could be regulated by TGF-β1 as aforementioned, we then determined the TGF-β1 level in the RSA patients. Consistently, TGF-β1 level was also decreased in the RSA patients compared with the normal pregnant women (Fig 6B). Therefore, TGF-β1 might be attributed to the distinct levels of Tim-3 on NK cells during normal and abnormal pregnancy. The reduced expression level of Tim-3 might be associated with the abnormal pregnancy.

## Discussion

We have shown here that dNK cells expressed higher level of Tim-3 than pNK cells and Tim-3 inhibited NK cytotoxicity toward trophoblasts via interaction with trophoblasts derived Galectin-9. In addition, we found Tim-3 was significantly increased in pNK during early pregnancy and Tim-3 abnormality may be associated with RSA. Our findings have demonstrated an additional mechanism by which Tim-3 serves as an immune regulator during pregnancy. More generally, whether Tim-3 regulates the roles of dNK including spiral arteriolar remodeling and trophoblasts invasion must be evaluated in the future.

Several studies have reported that Tim-3 expression was strikingly upregulated in peripheral immune cells including monocytes and T cells and abnormality of Tim-3 level may be associated with RSA and preeclampsia [19, 20]. Consistently, we have shown here Tim-3 mRNA and surface level on peripheral blood NK cells were significantly increased compared with non-pregnant control. Although Tim-3 level was varied among individuals, we found about 58% CD3^-^CD56^+^ pNK cells expressed Tim-3 and CD16^+^ NK cells expressed higher level of Tim-3 than CD16^-^ NK cells (data not shown). In addition, we found dNK cells were the primary Tim-3 positive cell type at the MFI and the level of Tim-3 expressed by dNK was obviously higher than pNK cells. It has been reported that a heterogeneous population of stage 3 NK precursor and mature stage 4 NK cells were present in uterine mucosa based on the expression of CD34, CD117 and CD94 [24]. Consistently, our results showed that the majority of freshly isolated dNK cells expressed CD94. However, Tim-3^+^ dNK expressed much more CD94 than Tim-3^-^ dNK cells. In addition, Tim-3^+^ dNK expressed higher levels of the activating receptors CD69. Therefore, the upregulation of Tim-3 during pregnancy may represent a specific activation status of dNK cells. Furthermore, it has been showed that Tim-3 marked highly functional NK cells with respect to both cytokines production and degranulation. Tim-3^+^ human pNK secreted more IFN-γ after IL-12 and IL-18 stimulation [14]. Tim-3^+^ dNK cells displayed higher IL-4 and lower TNF-α and perforin production [21]. In this paper, in order to further determine the effect of Tim-3 signal on basic cytokines expression of dNK, we cultured isolated dNK cells with low dose of IL-2 and found that blocking Tim-3 signal could significantly inhibit IFN-γ and TNF-α production. Although in the absence of Galectin-9 expressing target cells, Tim-3-Fc probably functioned via blocking NK autocrine Galectin-9 or other ligand of Tim-3. IFN-γ and TNF-α expressed by dNK cells significantly increased and played vital roles in successful pregnancy progress [25]. Ashkar, A. A. and colleagues showed that dNK-derived IFN-γ had essential role in the spiral arteriolar remodeling [26]. Recent report demonstrated dNK cells derived IFN-γ promoted immune tolerance and successful pregnancy by dampening inflammatory Th17 cells [27]. Embryo resorption in IL-10^-/-^ mice treated with LPS was correlated with elevated level of TNF-α and could be prevented by NK cells depletion or TNF-α blockade [7]. Therefore, the elevated expression of Tim-3 in dNK cells during early pregnancy might contribute to IFN-γ and TNF-α production and may regulate spiral arteriolar remodeling and local inflammatory response.

In contrast to pNK, dNK cells represent a unique NK cell subset which lack cytotoxicity but secrete proangiogenic factors and regulate trophoblast invasion [5, 28]. It has been reported that TGF-β1 can induce pNK to form dNK-like phenotype [29, 30]. Our results showed that TGF-β1 induced conversion from pNK to dNK followed by upregulation of Tim-3. Consistently, we showed that human decidua expressed TGF-β1 and dNK cell expressed higher level of TGF-βR I than pNK cells which may partially explain why dNK cells expressed higher level of Tim-3 than pNK cells. However, the precise mechanism and whether other factors such as intrauterine hormones are involved in the regulation of Tim-3 should be explored in the future study.

Despite some mechanisms for the non-cytotoxicity of dNK during pregnancy have been proposed, the regulation of dNK cell cytotoxicity toward trophoblasts in the MFI is still poorly understood. Our finding showed that blocking Tim-3 had no effect on NK cytotoxicity when K562 was used as target cells, which is consistent with other study [19]. However, the cytotoxicity of NK cells toward trophoblasts cell line HTR8 was significantly enhanced in the presence of Tim-3-Fc. Additionally, blocking Tim-3 signal increased IFN-γ production (data not shown) which was inconsistent with our results obtained from dNK. Probably, target cells HTR8 presence or not is the main reason for this difference. Moreover, Tim-3 signal may have distinct roles in dNK and pNK cells which need to be further determined. Most interesting, we found Galectin-9, the ligand of Tim-3, was differentially expressed by HTR8 and K562 cells. Galectin-9 was highly expressed by HTR-8 but not by K562. By using siRNA, we showed that Tim-3 inhibited NK cells cytotoxicity toward trophoblasts in Galectin-9 dependent pathway. By detecting CD107a, a newly defined marker of NK cells functional activity [31], we investigated the effect of Tim-3-Galectin-9 pathway on NK cells degranulation. As expected, CD107a level in NK cells significantly increased when co-cultured with HTR-8. Notably, blockage Tim-3-Galectin-9 pathway with Tim-3-Fc or Galectin-9 siRNA further upregulated CD107a expression. Meanwhile, we found dNK cell expressed low level CD107a than pNK cells in the presence of HTR-8 (data not shown). All these results indicated that Tim-3-Galectin-9 pathway inhibited NK cells cytotoxicity toward trophoblasts via regulating degranulation process.

In summary, our studies showed that by virtue of its up-regulation in NK cells, Tim-3 could render NK cells to secrete cytokines to sustain protective immunity and vascular remodeling meanwhile inhibit the cytotoxicity of NK cells toward trophoblasts to maintain local tolerance. Therefore, Tim-3 may play an important role in maintaining normal pregnancy and could be taken as a potential indicator to predict the risk of abortion during early pregnancy.
